# The Combined Supplementation of AZOMITE and Citric Acid Promoted the Growth, Intestinal Health, Antioxidant, and Resistance against *Aeromonas hydrophila* for Largemouth Bass, *Micropterus salmoides*

**DOI:** 10.1155/2023/5022456

**Published:** 2023-10-17

**Authors:** Yugui Zhang, Hongfei Huang, William T. H. Chang, Xiaoqin Li, Xiangjun Leng

**Affiliations:** ^1^National Demonstration Center for Experimental Fisheries Science Education, Shanghai Ocean University, Shanghai 201306, China; ^2^Centre for Research on Environmental Ecology and Fish Nutrition (CREEFN) of the Ministry of Agriculture and Rural Affairs, Shanghai Ocean University, Shanghai 201306, China; ^3^Shanghai Collaborative Innovation for Aquatic Animal Genetics and Breeding, Shanghai Ocean University, Shanghai 201306, China; ^4^Lytone Enterprise Inc., Taipei 221, China

## Abstract

Citric acid is an organic acid extensively used in feed industry, and AZOMITE is a hydrated aluminosilicate compound rich in rare earth elements and trace mineral elements. This study investigated the supplemental effects of AZOMITE and citric acid individual or in combination on the growth performance, intestinal microbiota, morphology, digestive enzyme activity, serum indexes, and disease resistance of juvenile largemouth bass. Six diets were designed, including the control diet (CON) and the five additive-supplemented diets with the addition of 4 or 8 g/kg citric acid (CA4, CA8), 3 g/kg AZOMITE (A3), and their combined addition as 4 g/kg citric acid + 1.5 g/kg AZOMITE) (C4A1.5) and 8 g/kg citric acid + 3 g/kg AZOMITE (C8A3). Juvenile largemouth bass with initial body weight of 22.01 ± 0.09 g were fed the six diets for 56 days. The results revealed that the combined addition of 4 g/kg citric acid and 1.5 g/kg AZOMITE (C4A1.5) increased weight gain by 7.99% (*P* < 0.05), and decreased feed conversion ratio by 0.07 (*P* < 0.05). The protein retention in the C4A1.5 group and the lipid retention in all additive-supplemented groups were significantly higher than those in the control group (*P* < 0.05). In serum, all additive-supplemented groups showed significantly higher glutathione peroxidase activity than the control group (*P* < 0.05). The activities of superoxide dismutase and catalase in the CA8, A3, C4A1.5, and C8A3 groups were significantly higher (*P* < 0.05), while the concentration of malondialdehyde was significantly lower than those in the control group (*P* < 0.05). Moreover, the total antioxidant capacity in the A3 and C4A1.5 groups, and lysozyme activity in the A3, C4A1.5, and C8A3 groups were significantly increased when compared to the control group (*P* < 0.05). In digestive enzyme, the protease activity in the A3, C4A1.5 groups, and amylase activity in the CA4, CA8, and C4A1.5 groups were significantly higher than those in the control group (*P* < 0.05). In intestinal microbiota, *Firmicutes* abundance was elevated in all additive groups, while the *Fusobacteriota* and *Plesiomonas shigelloides* abundance were decreased. In the intestinal histology, the CA8, A3, and C4A1.5 groups showed significantly higher villus height than the control group (*P* < 0.05). After the infection with *Aeromonas hydrophila*, the cumulative mortality of all additive-supplemented groups was significantly lower (*P* < 0.05), and the C4A1.5 group demonstrated the lowest mortality. In conclusion, the combined supplementation of 4 g/kg citric acid + 1.5 g/kg AZOMITE increased the growth, antioxidant, immune capacity, improved the intestinal morphology and microbial flora of juvenile largemouth bass, and promoted the resistance against *Aeromonas hydrophila* infection.

## 1. Introduction

Largemouth bass (*Micropterus salmoides*) is native to North America, and it is a typical carnivorous freshwater fish species with delicious flavor and without intermuscular bones. China is the largest producer in largemouth aquaculture with a production of 702,093 tons in 2021 (China Fishery Statistical Yearbook, 2022) [1]. In the past, the high-density farming and the unreasonable use of compound feed [2] increased the possibility and risk of disease occurrence. In the prevention and treatment of disease, antibiotics have been widely used [3]. However, the potential hazards posed by antibiotic use, such as drug resistance and residue, increased the potentially threatening to both humans and the environment [4–6]. In many countries, the use of antibiotics has been strictly restricted FAO [7]. Thus, the search for efficient and eco-friendly feed additives to enhance the immune function and disease-resistant ability of aquaculture animals has become a hot topic in the industry [8]. AZOMITE, a hydrated aluminosilicate mineral, is formed from volcanic eruption with plenty of natural mineral elements essential for the growth of animals and plants, along with rare earth elements such as lanthanum and actinium. AZOMITE has been used as mineral fertilizer and soil amendment in crop cultivation [9, 10]. In poultry, it was reported that dietary AZOMITE improved broiler's growth performance [11] and enhanced the mineral utilization such as calcium and phosphorus [12]. In aquaculture, AZOMITE supplementation in diets significantly enhanced the growth performance, immune capacity, and digestive function of tilapia (*Oreochromis niloticus* × *O.aureus*) [13] and grass carp (*Ctenopharyngodon idella*) [14], as well as improved the disease resistance of tilapia (*Oreochromis mossambicus*) [15].

As promising feed additives, organic acids and their salts have been reported some beneficial effects on aquatic animals [16], such as improving the growth performance [17] and immunity capacity [18], increasing digestive enzyme activity [19] and strengthening resistance against diseases [20], affecting the pH value in the intestine [21], and enhancing the utilization of minerals [22, 23]. Among these organic acids, citric acid (CA) is one of the most applied organic acids. Dietary citric acid or its salts has been reported to increase the digestive enzyme activity of hybrid tilapia (*Oreochromis niloticus* × *O. aureus*) [24], red drum (*Sciaenops ocellatus*) [19], turbot (*Scophthalmus maximus* L.) [25], and improve the growth performance of *Carassius auratus gibelio* [26], large yellow croaker (*Larimichthys crocea*) [27], rainbow trout (*Oncorhynchus mykiss*) [28], and their antioxidant capacity. Additionally, citric acid supplementation also enhanced phosphorus utilization in rainbow trout [29–31] and yellowtail (*Seriola quinqueradiata*) [23, 32], as well as increased the minerals utilization in various plant feedstuffs for rohu (*L. rohita*) [21].

Our previous studies have demonstrated that the addition of 2–3 g/kg AZOMITE in the diet significantly improved the growth performance, feed utilization, nonspecific immunity, *Aeromonas hydrophila* resistance, and intestinal morphology in juvenile largemouth bass [33]. However, the dietary application of citric acid has not been reported in largemouth bass. As citric acid could enhance the utilization of minerals, it is speculated that there may be a synergistic effect between citric acid and AZOMITE rich in minerals and rare earth elements. Therefore, this study investigated the supplemental effects of AZOMITE and citric acid individual or in combination on the growth performance, intestinal microbiota, morphology, digestive enzyme activity, serum antioxidant and immune indexes, and resistance against *Aeromonas hydrophila* infection of juvenile largemouth bass. Findings from this study will direct the development of efficient and eco-friendly feed additives for carnivorous fish.

## 2. Materials and Methods

### 2.1. Experimental Diets

A control diet was formulated to contain 490 g/kg crude protein and 130 g/kg crude lipid (CON), then citric acid and AZOMITE were added individually or in combination as follows: 4 g/kg citric acid (CA4), 8 g/kg citric acid (CA8), 3 g /kg AZOMITE (A3), 4 g/kg citric acid + 1.5 g/kg AZOMITE (C4A1.5), and 8 g/kg citric acid + 3 g/kg AZOMITE (C8A3) (Table 1). The inclusion levels of citric acid and AZOMITE referred to the findings in rainbow trout [28] and largemouth bass [33]. The AZOMITE was provided by Shanghai Lytone Biochemical Co., Ltd. (origin: AZOMITE® Mineral Products, Inc., USA), and the citric acid (AR 99.5% T) was purchased from Shanghai Macklin Biochemical Technology Co., Ltd.

The ingredients were ground and passed through a 60 mesh screen, mixed evenly, and then made into sinking pellets with a particle size of 2 mm using a single-screw extruder (LX-75 extruder, Longxiang Food Machinery Factory, Hebei, China). Granulation was performed at 85°C, and feed was dried to an approximate moisture content of 100 g per kilogram, then sealed for storage at room temperature.

### 2.2. Experimental Procedure

Largemouth bass were purchased from Fengmin Aquaculture Farm in Huzhou, Zhejiang Province (China). Initially, the fish were temporarily reared in indoor concrete pools for 2 weeks. Then, 450 healthy fish (22.01 ± 0.09 g) were selected and allotted in 18 indoor net cages (1.5 × 1.2 × 1.2 m), which were suspended in concrete pools (5 × 3 m). There were six treatment groups with three cages per group and 25 fish per cage. The water source was filtered with lake water (freshwater).

In the rearing period, the fish were fed twice daily (at 8:30 and 16:30) at a rate of 3%–5% of their body weight. All cages received the similar amount of feed. The cages were cleaned, and about 1/10 of the water was renewed once every 3 days to maintain the water quality. During the rearing period, the water quality was as follows: temperature 23–30°C, pH 6.5–7.3, dissolved oxygen >4 mg/L, salinity 0.5%–1.0%, nitrite <0.1 mg/L, and ammonia nitrogen 0.1–0.2 mg/L. The trial was carried out at Binhai Base of Shanghai Ocean University with a culture cycle of 56 days.

### 2.3. Samples Collection

Upon completion of 56 days of feeding, the fish were fasted for 24 hr, then the number of fish in each cage was counted, and the weight was measured. Weight gain (WG), feed conversion ratio (FCR), and survival were calculated from the above data.

Each cage contained three randomly selected fish for whole-body composition analysis, and blood was drawn from another three fish per cage via caudal vein after measuring body length and weight. The blood samples were centrifuged at 3,000 r min^−1^ for 10 mins, then the serum was collected for the determination of biochemical indicators.

The viscerosomatic index (VSI) and hepatosomatic index (HSI) of the three fish were calculated after blooding and dissection. Under sterile conditions, the intestinal tract was separated, and 1–2 cm of the anterior intestine was fixed in Bouin's solution for tissue observation, and the remaining parts were used for the measurement of intestinal digestive enzyme activity. Based on the growth performance, the CON, A3, CA8, C4A1.5, and C8A3 groups were selected for the intestinal microbial analysis, and three fish per cage were rapidly dissected in a sterile environment, then 1–2 cm of the intestine was sampled and stored in liquid nitrogen (the intestines of three fish were pooled as one sample) for the detection of intestinal microbial community composition.

### 2.4. Calculations Methods

#### 2.4.1. Growth Performance and Morphological Indices

The following formulas were used to calculate WG, FCR, survival, HSI, VSI, and CF (condition factor):

(1) $$\begin{array}{c} \text{WG}\left(\%\right)=\left[\text{final weight }\left(g\right)-\text{initial weight }\left(g\right)\right]/\text{initial weight }\left(g\right)\times 100\;, \end{array}$$

(2) $$\begin{array}{c} \text{FCR}=\text{dry feed consumed }\left(g\right)/\left[\text{final weight}\left(g\right)-\text{initial weight}\left(g\right)\right]\;, \end{array}$$

(3) $$\begin{array}{c} \text{Survival}\left(\%\right)=\text{final count}/\text{initial count}\times 100\;, \end{array}$$

(4) $$\begin{array}{c} \text{HSI}\left(\%\right)=\text{final liver weight}\left(g\right)/\text{final weight}\left(g\right)\times 100\;, \end{array}$$

(5) $$\begin{array}{c} \text{VSI}\left(\%\right)=\text{final visceral weight}\left(g\right)/\text{final weight}\left(g\right)\times 100\;, \end{array}$$

(6) $$\begin{array}{c} \text{CF}\left(\text{g }{\text{cm}}^{-3}\right)=\text{final weight}\left(\text{g}\right)/\text{final body length }{\left(\text{cm}\right)}^{3}\;. \end{array}$$

#### 2.4.2. Feed, Whole-Body Proximate Composition, and Nutrient Retention

AOAC [34] method was used to analyze diets and whole fish for moisture, ash, crude lipid, and crude protein contents. Crude protein was determined by Kjeldahl nitrogen method (Kjeltec 2300, Foss, Sweden) and moisture content by drying at 105°C. Chloroform–methanol method was used to determine the crude lipid content, and crude ash was detected by burning at 550°C in a Muffle furnace. Following is the calculation of protein retention (PR) and lipid retention (LR):

(7) $$\begin{array}{c} \text{PR}\left(\%\right)=\left[\text{final weight}\left(g\right)\times \text{crude protein of final fish }\left(\%\right)-\text{initial weight }\left(g\right)\times \text{crude protein of initial fish}\left(\%\right)\right]/\left[\text{feed intake}\left(g\right)\times \text{crude proteinof feed}\left(\%\right)\right]\times 100\;, \end{array}$$

(8) $$\begin{array}{c} \text{LR }\left(\%\right)=\;\left[\text{final weight }\left(g\right)\times \text{crude lipid of final fish }\left(\%\right)-\text{initial weight }\left(g\right)\times \text{crude lipid of initial fish}\left(\%\right)\right]/\left[\text{feed intake}\left(g\right)\times \text{crude lipid of feed}\left(\%\right)\right]\times 100\;. \end{array}$$

#### 2.4.3. Serum Biochemical Analysis

The serum biochemical indices included catalase (CAT, EC:1.11.1.6, colorimetric method), superoxide dismutase (SOD, EC:1.15.1.1, xanthine oxidase method), malondialdehyde (MDA, TBA colorimetric method), lysozyme (LYS, EC: 3.2.1.17, turbidimetric method), acid phosphatase (ACP, EC:3.1.3.2, colorimetric method), glutathione peroxidase (GSH-PX, EC:1.11.1.9, colorimetric method), total antioxidant capacity (T-AOC, colorimetric method), and total protein (TP, colorimetric method). All the measurements were performed using reagent kits produced by Shanghai Shunshi Biotechnology Co., Ltd.

#### 2.4.4. Intestinal Morphology, Digestive Enzyme Activity, and Microflora

The anterior intestine tissues were dehydrated through a series of ethanol solutions with increasing concentrations, followed by transparentizing in xylene and embedding in paraffin. Sections with thickness of 4 *μ*m were prepared using a microtome (Leika RM 2016, Germany). Hematoxylin and eosin (H&E) staining was performed, and the morphological structure of the intestinal tissue was observed and photographed using a microscope (Nikon YS100 Photomicrography System). Additionally, the height and width of villus and the thickness of the muscle layer were recorded (ImageJ).

The anterior gut samples were thawed at 4°C, followed by homogenization using nine times volume of 4°C physiological saline. After centrifuging for 15 min at (1,500 *g*, 4°C), the supernatant was collected to determine the digestive enzyme activity. The protease activity measurement referred to the method of Su et al. [35] by using 2% casein solution as the substrate, and the enzyme amount that decomposed casein to generate 1 *μ*g tyrosine per minute per milligram of tissue protein at pH 7.2 was defined as one protease activity unit (U). The amylase activity was determined using a reagent kit, and the hydrolysis of 10 mg starch per milligram of tissue protein at 37°C for 30 min was defined as one amylase activity unit (U).The total protein content was determined by a Coomassie brilliant blue method.

The intestine samples were sent to Shanghai Majorbio Bio-pharm Technology Co., Ltd. for intestinal microbiota analysis. DNA was extracted, and the PCR amplification was carried out, followed by high-throughput sequencing using the Illumina MiSeq platform. The primers V338F (5′-ACTCCTACGGGAGGCAGCAG-3′) and V806R (5′-GGACTACHVGGGTWTCTAAT-3′) were employed to amplify the 16S rRNA gene V3–V4 region. PCR conditions were as follows: predenaturation at 95°C for 3 min, followed by 29 cycles of 95°C for 30 s, 53°C for 30 s and 72°C for 45 s, then 72°C for 10 min. The obtained data were processed using the Majorbio Cloud Platform (https://www.majorbio.com) for operational taxonomic unit (OTU) clustering, and the composition and species abundance of microbial communities at the phylum and genus levels were calculated.

### 2.5. Challenge Test

The *Aeromonas hydrophila* used in this study was provided by the Aquatic Pathogen Collection Center of Shanghai Ocean University. The bacteria were cultured in Luria-Bertani (LB) broth at 30°C for 18 hr, then the required bacteria were obtained by centrifugation at 4°C for 10 min (3,500 *g*). A pretest was conducted before the challenge test to determine the LD50 by intraperitoneal injection of three concentrations (3 × 10^7^, 3 × 10^8^, and 3 × 10^9^ CFU/mL) at a dose of 0.01 mL/g body weight. Phosphate-buffered saline was used as the control, and the LD50 was determined to be 3 × 10^7^ CFU/mL (Bliss method). Ten fish per cage were selected after the sample collection and fed with the original diets for 3 days to restore normal metabolism. After 24 hr of fasting, the fish were intraperitoneally injected with a solution of *Aeromonas hydrophila* at 3 × 10^7^ CFU/mL (0.01 mL/g body weight). Then, the fish were observed continuously for 1 week after challenge, and the cumulative mortality rate was calculated.

### 2.6. Statistical Analysis

The experimental data were expressed as means ± standard deviation (SD). One-way analysis of variance (ANOVA) was performed using SPSS26.0 software to test for homoscedasticity and normal distribution of all data. Duncan's multiple range test was used for multiple comparisons between treatments with a significance level of *P* < 0.05.

## 3. Results

### 3.1. Growth Performance

The fish fed C4A1.5 diet had the highest WG and the lowest FCR with 7.99% increase in WG (*P* < 0.05) and 0.07 decrease in FCR (*P* < 0.05) compared to the CON. The CA8 group and A3 group also showed numerically higher WG (+5.47%, +4.25%) and lower FCR (−0.05, −0.03) than the CON (*P* > 0.05). There were no significant differences in survival, HSI, VSI, and CF among all the groups (*P* > 0.05) (Table 2).

### 3.2. Whole-Body Composition and Nutrient Retention

The whole-fish crude lipid content in the CA8, A3, and C4A1.5 groups, the PR in the C4A1.5 group and the LR in all additive-supplemented groups were significantly higher than those in the CON group (*P* < 0.05), but the crude protein and crude ash contents of all groups showed no significant differences (*P* > 0.05) (Table 3).

### 3.3. Serum Biochemical Analysis

In serum biochemical indices, all additive-supplemented groups showed significantly higher glutathione peroxidase (GSH-PX) activity than the CON group (*P* < 0.05). The activities of superoxide dismutase (SOD) and catalase (CAT) in the CA8, A3, C4A1.5, and C8A3 groups were significantly higher (*P* < 0.05), while the concentration of malondialdehyde (MDA) was significantly lower than those in the CON group (*P* < 0.05). Moreover, the total antioxidant capacity in the A3 and C4A1.5 groups, acid phosphatase activity in the CA4 and CA8 groups, and lysozyme activity in the A3, C4A1.5, and C8A3 groups were significantly increased as compared to the CON group (*P* < 0.05) (Table 4).

### 3.4. Intestinal Digestive Enzyme Activity

The protease activity in the A3, C4A1.5 groups, and amylase activity in the CA4, CA8, and C4A1.5 groups were significantly higher than those in the CON (*P* < 0.05) (Table 5).

### 3.5. Intestinal Microflora

**Figure 1:**
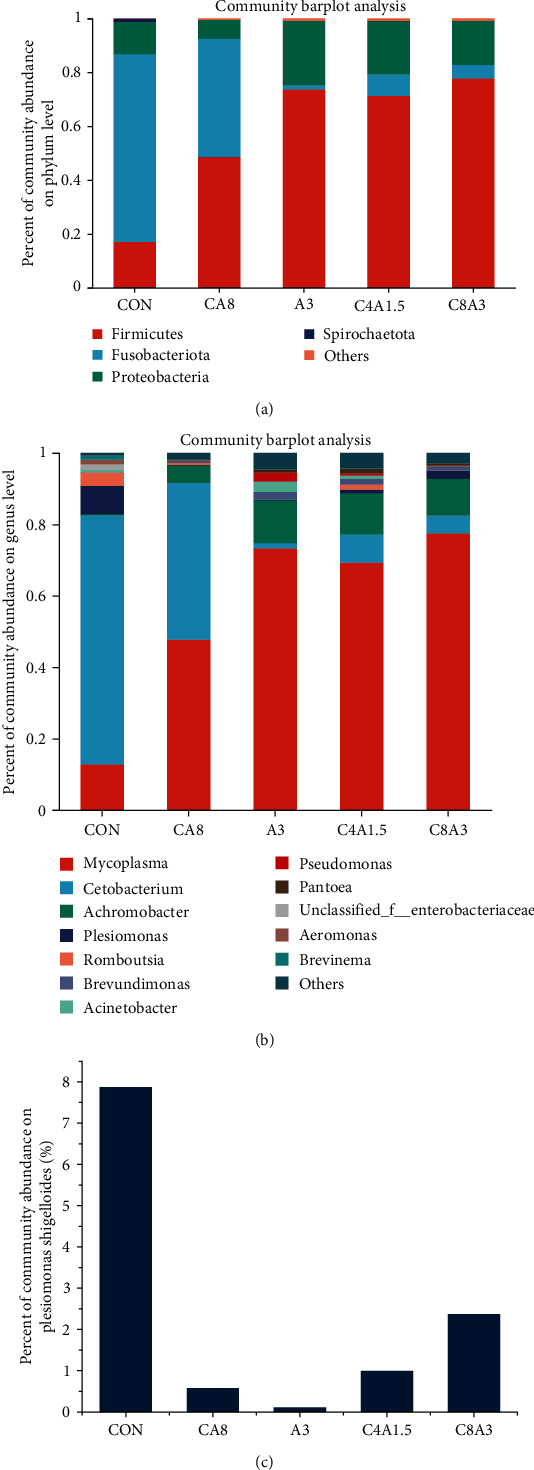
Relative abundance of intestinal bacteria of largemouth bass phylum level (a), genus level (b), and *Plesiomonas shigelloides* level (c).

At the phylum level (Figure 1(a)), the dominant bacteria were *Firmicutes*, *Fusobacteriota*, and *Proteobacteria*. The abundance of the three dominant phyla was 98.69%, 99.42%, 99.05%, 98.97%, and 99.08% in the CON, CA8, A3, C4A1.5, and C8A3 groups, respectively. As compared to the CON group, the proportion of *Firmicutes* increased and the proportion of *Fusobacteriota* decreased in the CA8, A3, C4A1.5, and C8A3 groups.

At the genus level (Figure 1(b)), the dominant species in the CON group were *Cetobacterium* (69.71%), *Mycoplasma* (12.78%), and *Plesiomonas* (7.87%). The dominant species in CA8, C4A1.5, and C8A3 were *Mycoplasma*, *Cetobacterium*, and *Achromobacter*, respectively. The dominant species in A3 were *Mycoplasma*, *Achromobacter*, and *Acinetobacter*.

In Figure 1(c)), the proportion of *Plesiomonas shigelloides* in the CON group was 7.87%, which decreased to 0.58%, 0.11%, 1%, and 2.37% in the CA8, A3, C4A1.5, and C8A3 groups, respectively.

### 3.6. Intestinal Morphology

**Figure 2:**
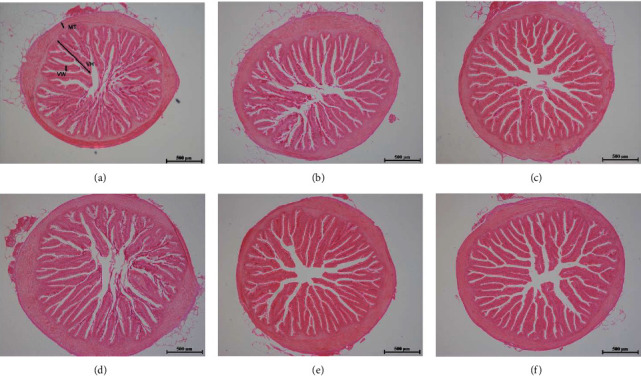
The intestinal structure of largemouth bass fed diets containing various levels of citric acid and (or) AZOMITE (40x). ((a)–(f)) represent the CON, CA4, CA8, A3, C4A1.5, and C8A3, respectively (MT, muscular thickness; VH, villus height; VW, villus width).

In Figure 2, it can be observed that the intestinal villus in the AZOMITE and citric acid mixture groups were arranged neatly and compactly with better clarity and integrity than the other groups. In Table 6, there was no significant difference in villus width and muscle thickness among all the groups. However, the CA8, A3, and C4A1.5 groups showed significantly higher villus height than the control group (*P* < 0.05).

### 3.7. Challenge Test

**Figure 3:**
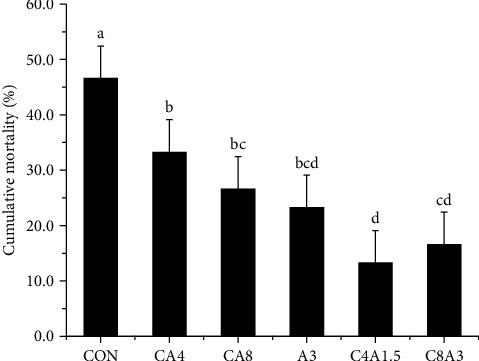
The cumulative mortality of largemouth bass after challenging against *A. hydrophila* during 7 days. Values are means ± SD (*n* = 10). Bars bearing with different letters are significantly different among treatments (*P* < 0.05).

After 7 days of infection with *Aeromonas hydrophila*, the cumulative mortality of largemouth bass in the CON group was 46.7%. The mortality in the CA4, CA8, A3, C4A1.5, and C8A3 groups were 33.3%, 26.7%, 23.33%, 13.33%, and 16.67%, respectively, significantly lower than that of the CON group (*P* < 0.05). Among them, the C4A1.5 group showed the lowest cumulative mortality (Figure 3).

## 4. Discussion

### 4.1. Growth Performance and Feed Utilization

Li et al. [28] reported that the addition of 8 and 12 g/kg of citric acid to diets significantly increased the WG and decreased the FCR of rainbow trout. In low-phosphorus diet, the supplemental citric acid (10 g/kg) significantly improved the growth performance of rainbow trout [29]. In large yellow croaker, the supplementation of 8 and 16 g/kg of citric acid to high-plant protein diets significantly enhanced the growth performance and protein retention [27]. Similarly, the improvement of growth performance by dietary citric acid has also been reported in red drum [19], red sea bream (*Pagrus major*) [22], *Carassius auratus gibelio* [26], and white shrimp (*Litopenaeus vannamei*) [35]. The promoting-effects of organic acids might be realized through reducing intestinal pH, stimulating digestive enzyme activity, inhibiting the growth of harmful intestinal microorganisms, and increasing the utilization of minerals [36]. However, dietary supplemental citric acid (0–30 g/kg) did not significantly affect the growth performance of turbot [25]. In the present study, adding 4 or 8 g/kg of citric acid to diet just showed an increasing trend in the growth performance (*P* > 0.05), which may be related to the low-addition level of citric acid, and further study is needed to investigate dietary effects of citric acid on largemouth bass with graded citric acid level.

AZOMITE is a natural hydrated aluminosilicate rich in rare earth elements. According to the study of Xu et al. [33], adding 2.0 g/kg of AZOMITE to the diet of juvenile largemouth bass increased WG by 11.2% and reduced FCR by 0.1. The growth-promoting effects of AZOMITE have also been observed in tilapia [13], grass carp [14], and white shrimp [37]. The rich rare earth and trace elements in AZOMITE, such as La, Ce [38–40], have antibacterial properties and may enhance the digestive and absorption capacity of animal, thus positively affecting growth performance. In this study, the addition of 3.0 g/kg of AZOMITE just numerically improved the growth performance of largemouth bass, which may be connected with the growth stages, diet composition, and aquaculture environments.

Xun et al. [41] once reported that adding rare earth citrate to diet increased the nutrient digestibility of sheep, and the dietary rare earth citrate also improved the growth performance of broilers [42]. In this study, the combination of 4 g/kg of citric acid and 1.5 g/kg of AZOMITE significantly increased WG and decreased FCR. This promoting effect may be due to the synergistic effect of citric acid and AZOMITE, which increases the utilization of minerals, stimulates the secretion of digestive juice, enhances the activity of protease and amylase. However, the high inclusion of citric acid + AZOMITE (8+ 3 g/kg) did not significantly improved the growth of largemouth bass, which may be due to the antagonistic effect of the excessive supplementation.

### 4.2. Antioxidant Capacity

Fish's antioxidant capacity plays an important role in their health [43]. Excessive reactive oxygen species (ROS) can cause DNA hydroxylation, lipid peroxidation, and other effects leading to cell apoptosis and compromised immunity [44, 45]. Antioxidant systems can control the ROS level and maintain the balance in body, thus enhancing immunity [46].

Superoxide dismutase (SOD) can clear superoxide radicals by catalyzing superoxide radicals (O^2−^) to form hydrogen peroxide and water, reducing the synthesis of OH radicals [44]. During the reduction of oxygen in the body, hydrogen peroxide is also produced, and excessive hydrogen peroxide will threaten the body's homeostasis [47], while catalase (CAT) and glutathione peroxidase (Gpx) can clear excess hydrogen peroxide. Malondialdehyde (MDA) is a product of lipid peroxidation caused by excessive ROS, which may negatively affect the activity of proteins in the body [48]. Total antioxidant capacity (T-AOC) directly reflects the total antioxidant capacity of the organism [49]. Therefore, the activity of SOD, CAT, the concentration of GPX, MDA, and T-AOC are important indicators of reflecting antioxidant capacity [50].

Dietary supplementation of citric acid can help to improve the antioxidant capacity of fish. For example, dietary citric acid has been reported to promote the antioxidant capacity and reduce MDA content in rainbow trout [28], turbot [51], and large yellow croaker [27]. In this study, the addition of 8 g/kg citric acid, rather than 4 g/kg, significantly increased the activity of SOD, CAT, and GPx and reduced MDA concentration. Thus, the positive effect of citric acid is closely related to the inclusion level.

Rare earth elements also have positive effects on the body's antioxidant capacity. In tilapia, [13], grass carp [14] and white shrimp [37], the supplemental AZOMITE (2, 4 g/kg) significantly increased serum SOD activity. Xu et al. [33] also reported that dietary AZOMITE (1–6 g/kg) increased the serum antioxidant enzyme activity (SOD/CAT) of largemouth bass at different levels. In this study, the addition of 3 g/kg of AZOMITE significantly increased SOD, CAT, GPx activities, and T-AOC level, and reduced the MDA concentrations in serum.

In the present study, the combined supplementation of citric acid and AZOMITE (C4A1.5, C8A3) significantly increased serum SOD, CAT, GPx activities, and T-AOC levels, and reduced the MDA concentrations. It is noteworthy that the SOD and CAT activities in the two combined groups were significantly higher than those in the two citric acid groups, and numerically higher than those in the AZOMITE group. Maybe a synergistic effect between citric acid and rare earth elements in AZOMITE was produced, further enhancing the body's antioxidant capacity. However, the mechanism is unclear and needs further study.

### 4.3. Intestinal Microbiota

The intestinal microorganism is not only an important defense line in fish immune systems, but also participates in nutrient absorption [52]. Various intrinsic or extrinsic factors, such as fish species, development stage, water environment, and food would affect the composition of intestinal microbiota [53]. The present study indicated that the dominant intestinal microbiota (at the phylum level) of largemouth bass were *Firmicutes*, *Fusobacteriota*, and *Proteobacteria*, which accounted for more than 98% of total bacteria in all groups. At the genus level, the dominant species were *Cetobacterium* (69.71%), *Mycoplasma* (12.78%), and *Plesiomonas* (7.87%). In other groups except the A3 group, the dominant genus were *Mycoplasma*, *Cetobacterium*, and *Achromobacter*. Such results were consistent with the reports by Yang et al. [54], He et al. [55], and Zhou et al. [56].

At the phylum level, the addition of citric acid and AZOMITE did not affect the dominant species composition in the intestinal microbiota. However, at the genus level, the abundance of *Mycoplasma* was significantly increased in all additive groups compared to the control group. There are few reports on the role of *Mycoplasma* in the intestinal microbiota of fish. Although some species of *Mycoplasma* are pathogenic, previous studies have shown that certain species of this genus are harmless commensals in the natural intestinal microbiota. For example, *Mycoplasma* is the dominant species in the normal intestinal microbiota of *Gillichthys mirabilis* [57] and wild salmon (*Salmon*) [58].

It has been reported that *Plesiomonas shigelloides* is a conditionally pathogenic bacterium that causes significant loss to aquatic animals [59]. In this study, *Plesiomonas shigelloides* accounted for 7.87% in the CON and 0.58%, 0.11%, 1%, and 2.37% in the CA8, A3, C4A1.5, and C8A3 groups, respectively, which indicated that citric acid and AZOMITE addition inhibited the growth and colonization of this bacterium in the intestine, possibly enhancing the host's disease resistance.

### 4.4. Intestinal Morphology and Digestive Enzyme Activity

The intestinal morphology directly reflects the health of the intestine, and the digestive enzyme activity is an important indicator reflecting the ability to digest nutrients. Dai et al. [25] found that the intestinal absorption area of turbot was increased by the supplementation of 15 g/kg citric acid. Huang et al. [60] reported that dietary calcium sulfate increased the villus height and width of largemouth bass. Dietary citric acid (10 g/kg) has also been reported to significantly improve the digestive enzyme activity in intestine of tilapia [13]. Similar results were obtained in white shrimp, rainbow trout and red drum [19, 28, 35]. In this study, the villus height in the CA8 group and the amylase activity in the CA4 and CA8 groups increased, which may be related to citric acid's ability to reduce the pH and stimulate the secretion of intestine.

Rare earth elements also have the function of improving intestinal structure. Xu et al. [33] found that dietary AZOMITE increased the intestinal villus height and digestive enzyme activity of largemouth bass [33], and Liu et al. [13] reported the similar results in tilapia. In this study, the villus height and protease activity in the A3 and C4A1.5 groups were also significantly increased, the improvement of intestinal structure and digestive enzyme activity may be related to the improvement of the intestinal microbial community and the synergistic effect of acid and rare earth elements. However, the C8A3 group did not present the promoting effects on villus height and protease activity, which may be due to the antagonistic effect of the excessive supplementation.

### 4.5. Immunological Indices and Disease Resistance

Acid phosphatase (ACP) and lysozyme (LZM) are important indicators for the immune function [61]. Su et al. [35] reported that dietary supplementation of citric acid significantly increased the lymph lysozyme activity and the survival after *Vibrio parahaemolyticus* infection. Zhang et al. [26] found that citric acid and malic acid enhanced the alkaline phosphatase activity of *Carassius auratus gibelio* and increased the gene expression of *IL-1β*. Adding 2–5 g/kg AZOMITE to the diet of largemouth bass also significantly increased lysozyme activity and reduced cumulative mortality after *Aeromonas hydrophila* challenge [33]. Similar findings in AZOMITE have been reported in tilapia [15] and white shrimp [37]. In this study, the ACP activity in the CA4, CA8 groups, the LZM activity in the A3, C4A1.5, and C8A3 groups were significantly increased, and the cumulative mortality after *Aeromonas hydrophila* infection in all citric acid and (or) AZOMITE groups was decreased, when compared to the CON group. Such results indicated that the immunity and disease resistance were promoted by the supplementation. Specially, the lowest cumulative mortality was observed in the C4A1.5 group, indicating a synergistic effect of citric acid (4 g/kg) and AZOMITE (1.5 g/kg) in enhancing disease resistance. This may be due to the anions of citric acid chelating the active components in AZOMITE in the intestine, stimulating the immune function, and enhancing the resistance to bacterial infection.

## 5. Conclusion

In conclusion, the combined supplementation of citric acid and AZOMITE increased the growth, antioxidant, immune capacity, improved the intestinal morphology and microbial flora, and promoted the disease resistance against *Aeromonas hydrophila* infection. A dietary dosage of 4 g/kg citric acid + 1.5 g/kg AZOMITE are recommended for juvenile largemouth bass.

## Figures and Tables

**Table 1:** Ingredients and proximate composition of experimental diets (air-dry basis, g/kg).

Ingredients^a^	CON	CA4	CA8	A3	C4A1.5	C8A3
Fish meal	350.0	350.0	350.0	350.0	350.0	350.0
Soybean meal	100.0	100.0	100.0	100.0	100.0	100.0
Soy protein concentrate	120.0	120.0	120.0	120.0	120.0	120.0
Cottonseed protein concentrate	50.0	50.0	50.0	50.0	50.0	50.0
Wheat flour	125	121	117	122	119.5	114
Pork meal	60.0	60.0	60.0	60.0	60.0	60.0
Other ingredients^b^	175.0	175.0	175.0	175.0	175.0	175.0
Vitamin and mineral premix^c^	20.0	20.0	20.0	20.0	20.0	20.0
Azomite	0	0	0	3	1.5	3.0
Citric acid	0	4.0	8.0	0.0	4.0	8.0
Total	1000.0	1000.0	1000.0	1000.0	1000.0	1000.0
Proximate composition						
Crude protein	491.3	490.2	495.8	494.8	493.9	495.3
Crude lipid	129.5	130.2	129.2	128.8	129.8	129.3
Ash	97.3	97.4	100.4	105.0	103.6	104.2
Moisture	108.7	104.6	107.5	105.1	104.5	103.0

^a^The feedstuffs were purchased from Hanbei Feed Company. The crude protein contents of fish meal, soybean meal, soy protein concentrate, cottonseed meal, wheat flour, pork meal, and wheat gluten meal were 672, 442, 654, 600, 144, 740, and 752 g/kg, respectively. ^b^Other ingredients (g/kg) included: wheat gluten meal (40.0), squid oil (40.0), fish oil (25.0), soybean oil (25.0), and soybean lecithin (25.0). ^c^The vitamin and mineral premix (mg or IU kg^−1^ of feed) included: VA, 11,000 IU; VD_3_, 3,100 IU; VC, 110 mg; VE, 140 IU; VB_1_, 18 mg; VB_2_, 20 mg; VB_6_, 6 mg; VB_12_, 0.16 mg; pantothenic acid, 60 mg; niacin, 60 mg; folic acid, 7 mg; biotin, 0.5 mg; inositol, 500 mg; I, 1 mg; Co, 1.2 mg; Cu, 7.6 mg; Fe, 62 mg; Zn, 71 mg; Mn, 10.4 mg; Se, 0.3 mg; and Mg, 160 mg.

**Table 2:** Effects of dietary AZOMITE and citric acid on growth performance and physical indices of largemouth bass.

Index	CON	CA4	CA8	A3	C4A1.5	C8A3
IBW/g	22.03 ± 0.06	22.00 ± 0.01	22.07 ± 0.04	22.03 ± 0.12	22.10 ± 0.19	21.96 ± 0.01
FBW/g	79.22 ± 1.06^a^	82.35 ± 0.14^ab^	82.35 ± 1.30^ab^	81.65 ± 2.10^ab^	83.8 ± 1.4^b^	80.56 ± 2.89^ab^
WG/%	260.11 ± 4.83^a^	274.30 ± 0.65^ab^	274.33 ± 5.90^ab^	271.16 ± 9.53^ab^	280.89 ± 6.37^b^	266.18 ± 13.14^ab^
FCR	0.99 ± 0.02^a^	0.94 ± 0.03^ab^	0.94 ± 0.01^ab^	0.95 ± 0.03^ab^	0.92 ± 0.02^b^	0.97 ± 0.05^ab^
Survival/%	100	100	100	100	100	100
VSI/%	7.15 ± 0.5	7.10 ± 0.43	7.00 ± 0.08	7.07 ± 0.34	7.08 ± 0.85	7.07 ± 0.65
HSI/%	1.25 ± 0.26	1.23 ± 0.15	1.26 ± 0.19	1.30 ± 0.13	1.25 ± 0.19	1.30 ± 0.09
CFg/cm^−3^	2.10 ± 0.09	2.09 ± 0.09	2.10 ± 0.14	2.24 ± 0.16	2.13 ± 0.07	2.09 ± 0.12

Values in the same row with different superscripts alphabets indicate significant differences (*P* < 0.05), the same as below. IBW, initial body weight; FBW, final body weight; WG, weight gain; FCR, feed conversion ratio; HSI, hepatosomatic index; VSI, viscerosomatic index; and CF, condition factor.

**Table 3:** Effects of dietary AZOMITE and citric acid on whole-body composition and nutrient retention of largemouth bass.

Items	CON	CA4	CA8	A3	C4A1.5	C8A3
Moisture (%)	71.86 ± 0.06	71.22 ± 0.46	72.01 ± 0.64	71.00 ± 0.87	70.93 ± 0.08	71.83 ± 0.37
Crude protein (%)	17.06 ± 0.01	17.52 ± 0.07	17.36 ± 0.01	17.62 ± 0.15	17.91 ± 0.12	16.96 ± 0.10
Crude lipid (%)	4.09 ± 0.08^a^	4.62 ± 0.01^ab^	5.03 ± 0.12^bc^	5.34 ± 0.12^c^	4.95 ± 0.09^bc^	4.67 ± 0.54^ab^
Crude ash (%)	4.10 ± 0.06	4.39 ± 0.34	4.10 ± 0.18	4.31 ± 0.09	3.96 ± 0.13	4.48 ± 0.53
Protein retention (%)	35.60 ± 0.30^a^	38.94 ± 2.03^ab^	38.48 ± 0.17^ab^	38.77 ± 3.24^ab^	41.68 ± 2.91^b^	36.68 ± 0.85^ab^
Lipid retention (%)	32.44 ± 1.46^a^	40.13 ± 0.08^b^	44.74 ± 0.89^bc^	47.77 ± 2.81^c^	44.86 ± 2.37^bc^	40.04 ± 3.86^b^

**Table 4:** Effects of dietary AZOMITE and citric acid on hematology and biochemical parameters of largemouth bass.

Items	CON	CA4	CA8	A3	C4A1.5	C8A3
SOD (u/ML)	96.37 ± 7.43^a^	100.2 ± 4.84^a^	118.89 ± 4.12^b^	125.25 ± 3.58^bc^	132.68 ± 9.38^c^	130.53 ± 1.59^c^
CAT (U/ml)	7.26 ± 0.27^a^	7.38 ± 0.48^a^	8.36 ± 0.26^b^	9.27 ± 0.66^b^	9.12 ± 0.01^b^	8.94 ± 0.13^b^
GSH-PX (U/ml)	782.8 ± 6.6^a^	867.7 ± 37.0^b^	868.8 ± 35.4^b^	945.8 ± 23.7^c^	1015.6 ± 44.2^c^	975.0 ± 34.8^c^
T-AOC (U/ ml)	23.31 ± 3.31^a^	25.78 ± 5.06^ab^	33.67 ± 2.62^abc^	38.23 ± 3.49^c^	35.89 ± 5.41^bc^	30.09 ± 4.19^abc^
MDA (nmol/ml)	4.60 ± 1.00^a^	4.51 ± 1.00^a^	2.92 ± 0.38^b^	2.17 ± 0.19^b^	1.81 ± 0.44^b^	1.55 ± 0.31^b^
TP (gprot/L)	30.05 ± 1.61^ab^	33.48 ± 0.81^ab^	28.72 ± 3.23^a^	30.81 ± 0.81^ab^	31.38 ± 0.81^ab^	33.38 ± 1.75^b^
ACP (U/ml)	0.134 ± 0.004^a^	0.156 ± 0.024^b^	0.164 ± 0.005^b^	0.153 ± 0.006^ab^	0.149 ± 0.003^ab^	0.139 ± 0.011^a^
LZM (ug/ml)	4.88 ± 0.38^a^	4.88 ± 0.17^a^	4.47 ± 0.09^a^	6.42 ± 0.34^b^	6.80 ± 0.26^b^	6.39 ± 0.33^b^

SOD, superoxide dismutase; CAT, catalase; GSH-PX, glutathione peroxidase; T-AOC, total antioxidant capacity; MDA, malondialdehyde; TP, total protein; ACP, acid phosphatase; and LZM, lysozyme.

**Table 5:** Effects of dietary AZOMITE and citric acid on digestive enzyme activities of largemouth bass.

Items	Protease activity/(U/mg protein)	Amylase activity/(U/mg protein)
CON	98.62 ± 4.69^a^	0.28 ± 0.02^a^
CA4	106.16 ± 1.21^ab^	0.33 ± 0.01^b^
CA8	107.62 ± 1.14^ab^	0.35 ± 0.01^b^
A3	109.34 ± 6.54^b^	0.325 ± 0.01^ab^
C4A1.5	109.88 ± 2.72^b^	0.33 ± 0.02^b^
C8A3	106.27 ± 1.72^ab^	0.32 ± 0.05^ab^

Values in the same column with different superscripts alphabets indicate significant differences (*P* < 0.05), the same as below.

**Table 6:** Effects of dietary AZOMITE and citric acid on intestinal morphology of largemouth bass.

Items	Villus height (*μ*m)	Villus width (*μ*m)	Muscle thickness (*μ*m)
CON	825.00 ± 17.35^a^	91.27 ± 0.49	132.33 ± 3.06
CA4	852.67 ± 20.65^ab^	91.82 ± 2.56	135.33 ± 2.52
CA8	866.33 ± 7.37^b^	93.47 ± 2.90	134.00 ± 2.65
A3	875.67 ± 31.63^b^	93.77 ± 1.80	134.67 ± 3.21
C4A1.5	867.00 ± 18.73^b^	94.15 ± 2.60	135.60 ± 2.12
C8A3	851.00 ± 9.90^ab^	92.77 ± 2.74	132.67 ± 3.06

## Data Availability

All data generated or analyzed during this study are included in this article.
